# Microneedle-mediated nanomedicine to enhance therapeutic and diagnostic efficacy

**DOI:** 10.1186/s40580-024-00421-w

**Published:** 2024-04-18

**Authors:** Yuyang Zuo, Rujie Sun, Nuala Del Piccolo, Molly M. Stevens

**Affiliations:** 1 Department of Materials, Department of Bioengineering, and Institute of Biomedical Engineering, Imperial College London, London, SW7 2AZ UK; 2 Department of Physiology, Anatomy and Genetics, Department of Engineering Science, and Kavli Institute for Nanoscience Discovery, University of Oxford, Oxford, OX1 3QU UK

**Keywords:** Microneedle, Nanomedicine, Drug delivery, Diagnostics, Therapeutics

## Abstract

Nanomedicine has been extensively explored for therapeutic and diagnostic applications in recent years, owing to its numerous advantages such as controlled release, targeted delivery, and efficient protection of encapsulated agents. Integration of microneedle technologies with nanomedicine has the potential to address current limitations in nanomedicine for drug delivery including relatively low therapeutic efficacy and poor patient compliance and enable theragnostic uses. In this Review, we first summarize representative types of nanomedicine and describe their broad applications. We then outline the current challenges faced by nanomedicine, with a focus on issues related to physical barriers, biological barriers, and patient compliance. Next, we provide an overview of microneedle systems, including their definition, manufacturing strategies, drug release mechanisms, and current advantages and challenges. We also discuss the use of microneedle-mediated nanomedicine systems for therapeutic and diagnostic applications. Finally, we provide a perspective on the current status and future prospects for microneedle-mediated nanomedicine for biomedical applications.

## Introduction

Nanomedicine and microneedles are emerging technologies that have significant potential to advance healthcare [1, 2] by enhancing medication efficacy, improving patient experience, achieving earlier disease detection, and enabling personalized healthcare [3].

As an application of nanotechnology, nanomedicine harnesses the unique properties of nanoscale materials for healthcare innovations; it aims to transform disease monitoring, diagnosis, and therapy [4]. The concept of nanotechnology was first introduced by Nobel Prize laureate Richard Feynman in his famous lecture “There’s Plenty of Room at the Bottom” in 1959 [5], in which he considers materials and devices at the nanoscale—typically at dimensions below 100 nm [6]. Nanomedicine has promise in many applications, including drug delivery [7], imaging [8, 9], sensing [10], and tissue engineering [11]. Nanoparticles are widely applied in nanomedicine due to their unique properties at the nanoscale, which make them highly advantageous for various biomedical applications. For example, nanoparticles [12] as drug carriers can accurately deliver drugs to a target site by carrying therapeutic agents [13] or through functionalization with therapeutic ligands [14] such as antibodies [15], peptides [16], or polymers [17]. Compared with conventional delivery systems, nanoparticles exhibit several advantages, including sustained release, efficient protection of encapsulated materials, and targeting function [18]. Nanoparticles used as contrast agents can also enable highly sensitive and specific imaging modalities: for example, quantum dots [19] can enhance imaging contrast in medical diagnostics. In recent years, several administration methods have been developed to deliver nanoparticles to targeted sites, including intravenous injection [20], oral administration [21], intratumoral injection [22], transdermal delivery [23], inhalation [24], intraperitoneal injection [25] and ocular delivery [26].

Despite numerous achievements, the applications of nanoparticles still face challenges, including effective administration methods, safety concerns, and regulatory considerations [27]. Nanoparticles are typically administered by hypodermic needles, which induce patient discomfort and thus reduce patient compliance [28]. Although nanoparticles can be designed for targeted delivery, their efficacy can be affected by several factors such as the enhanced permeability and retention (EPR) effect and resistance mechanisms in tumor cells [29]. Nanoparticles can also be quickly cleared from the bloodstream by the immune system to the liver [30], which can significantly reduce drug concentration and thus therapeutic effect. In addition, biological barriers—including the endothelium, skin, mucus, and cell membranes[31]—can obstruct the penetration of nanoparticles into targeted sites.

Microneedle technologies have received increasing attention in recent years due to their minimally invasive design and dimensions, which avoid triggering nerves and thus minimize patient pain [32]. Microneedle-based delivery of nanoparticles can support transport across most physical barriers inside the body, precisely reach targeted sites with high efficiency, avoid clearance by the immune system, and minimize serious adverse effects. Additionally, this technique can improve sustainability by reducing dosing frequency and increase cost effectiveness by minimizing the production of sharp medical waste in the form of needles and syringes [33]. Moreover, microneedle patches provide a less painful and more convenient way to administer nanoparticles with minimal expert supervision [2, 34], which could increase patient acceptability and adherence.

**Fig. 1:**
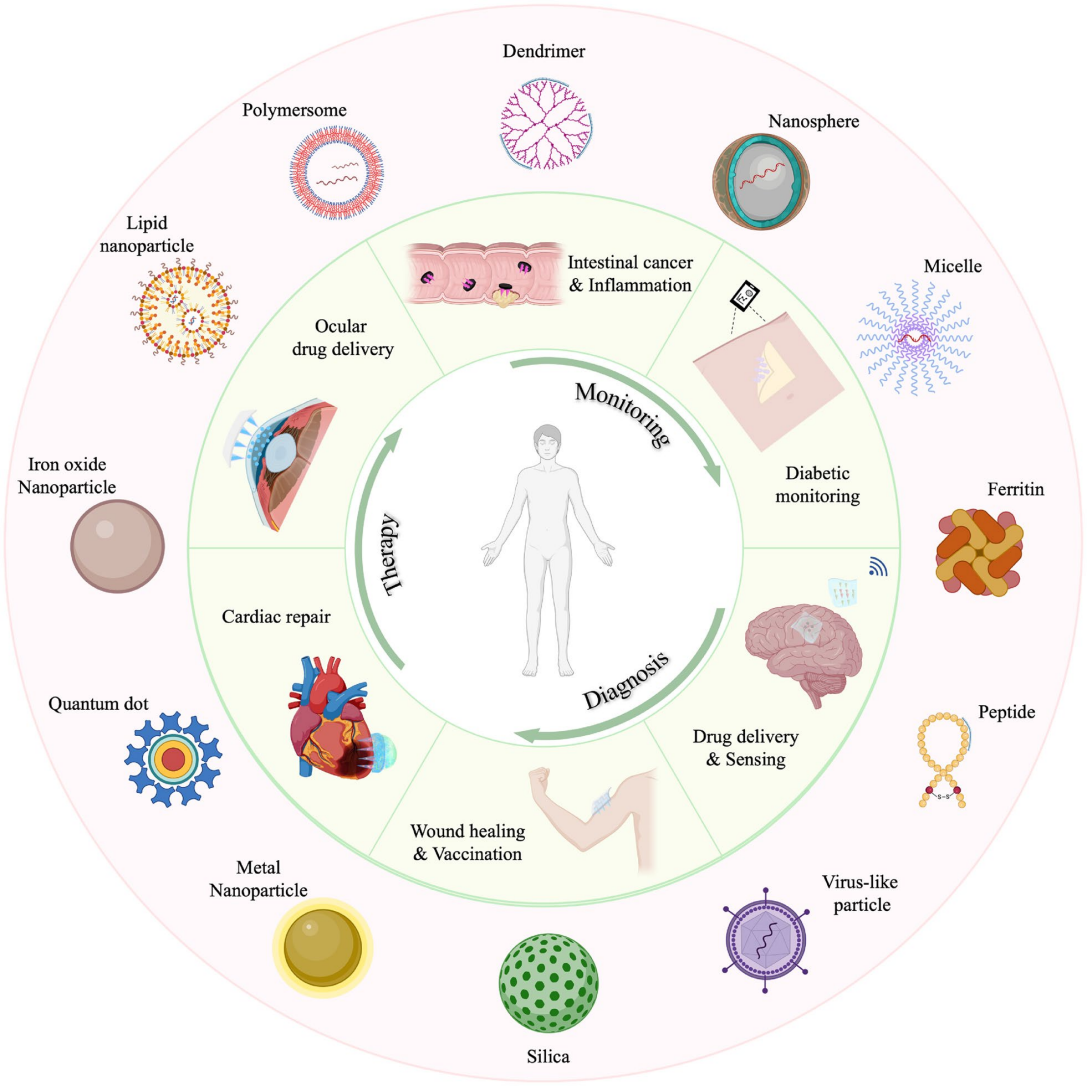
Diverse nanomedicine systems can be integrated with microneedle technologies for disease monitoring, diagnosis, and therapy

Due to the respective advantages of nanomedicine and microneedle technologies, microneedle-based delivery of nanoparticles has attracted considerable attention over the past few decades (Fig. 1). The purpose of this Review is to explore the potential mechanisms and applications of technologies which combine microneedle and nanoparticle systems. We provide a brief overview of the current state of nanoparticle and microneedle design, development, and applications. Next, we summarize the potential applications of microneedle-assisted nanomedicine systems. Finally, we discuss current challenges and future developments in microneedle-assisted nanomedicine strategies.

## Current development of nanomedicine

### Fundamentals of design and formulation

Nanomedicine is an emerging field that applies the principles of nanotechnology to the medical field to revolutionize human healthcare. Specifically, nanomedicine utilizes the properties and structures of nanoscale materials, such as nanoparticles, to achieve a wide range of therapeutic and diagnostic applications. Efforts to use tiny particles to improve drug delivery can be traced back to the early twentieth century, but the potential of nanomedicine was realized in the late twentieth century. Currently, approximately 50 nanomedicine therapies for cancers and other diseases have been approved by the US Food and Drug Administration (FDA).

**Fig. 2:**
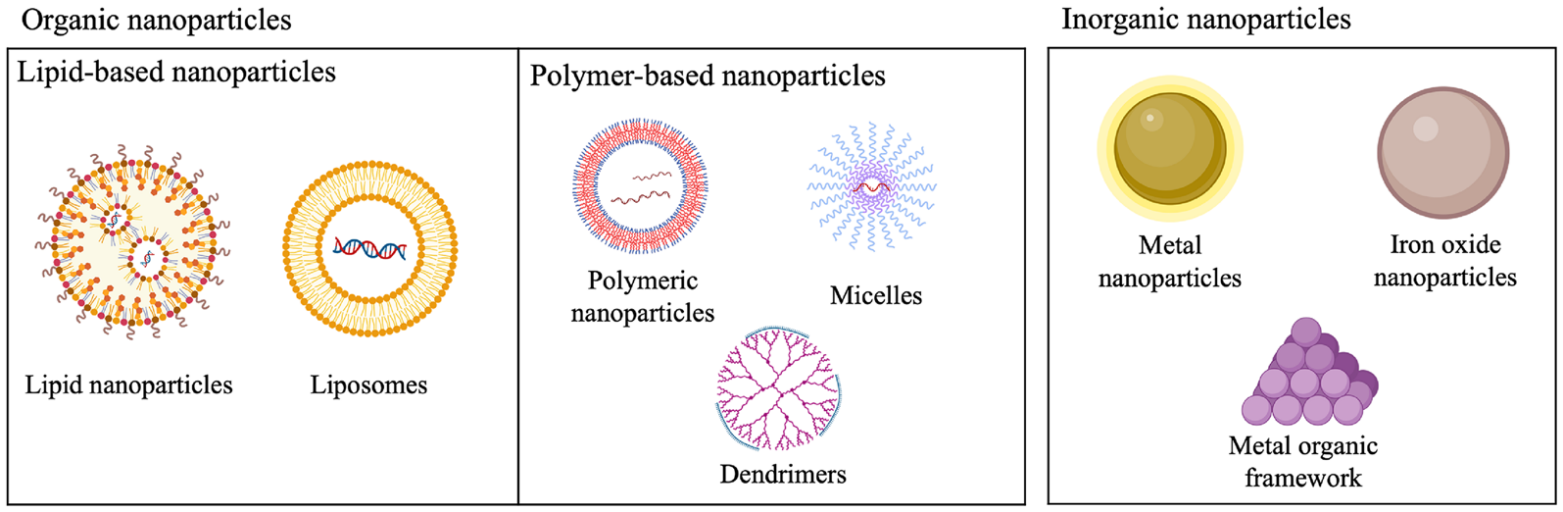
Types of nanoparticles that hold promise for biomedical applications, including lipid-based nanoparticles, polymer-based nanoparticles, and inorganic nanoparticles

One of the primary advantages of nanomedicine is the ability to enhance drug delivery for therapeutic applications. Traditional drug delivery methods face several barriers, including poor solubility, lack of targeting function, and potential side effects. Larger than nanoscale sized materials can be hindered by problems such as in vivo instability, poor bioavailability, and poor absorption in the body [35]. In comparison, nanoparticles can deliver drugs to specific sites, thus reducing side effects and improving drug efficacy [36]. Nanoparticles have also shown promise in applications outside drug delivery: for example, they can be used as contrast agents in imaging technologies to enhance the precision and resolution of images or employed for photothermal and photodynamic therapy (PDT) to inhibit tumor growth. In this section, we will discuss the design and formulation of multiple nanomaterial systems, including organic and inorganic nanoparticles (Fig. 2).

#### Organic nanomaterials

Organic nanomaterials are formed via covalent or noncovalent assemblies of organic molecules. Unlike inorganic nanomaterials, which are mainly based on metals and metallic derivations, organic nanomaterials derive unique structures and properties from the versatile chemistry of natural or synthetic molecules. They have several advantages—including tunability, biocompatibility, and biodegradability—which make these materials suitable for a wide range of biomedical applications. A summary of organic nanomaterials and their applications is shown in Table 1, where lipid- and polymer-based nanomaterials are representative examples.

**Table 1:** Summary of common organic nanomaterials and their applications

Nanomaterials	Materials	Applications	Refs.
Liposomes	HSPC, cholesterol, SP-PEG3400-DSPE, mPEG2000-DSPE	Brain-targeted drug delivery	[37]
	DPPC, DSPE-mPEG, DSPE-PEG-Mal, cholesterol	Molecular imaging	[38]
	DOTAP, cholesterol, DP7-C	mRNA vaccine	[39]
Lipid nanoparticles	SOPC, PEG-DMG, cholesterol, DLin-KC2-DMA	Gene therapy	[40]
	ALC-0315, ALC-0159, DSPC, cholesterol	Pfizer Covid-19 vaccine	[41]
	SM-102, PEG2000-DMG, DSPC, cholesterol	Moderna Covid-19 vaccine	[41]
Polymeric nanoparticles	PEI, PEG2000, hyperbranched bis-MPA polyester	Gene editing therapy	[42]
	Chitosan, ascorbic acid, penta-sodium tripolyphosphate	Cervical cancer therapy	[43]
	Poly (CBA-co-4-amino-1-butanol) (pABOL)	Self-amplifying mRNA delivery	[44]
Dendrimers	PAMAM, TNBSA	Breast cancer therapy	[45]
	PPI-m OS G4, Ara-CTP	Drug delivery	[46]
	PLLD-G4, HPG-C18	Gene delivery, Drug delivery	[47]
Micelles	GE11 peptide, SPION, chitosan oligosaccharide	MRI diagnosis	[48]
	mPEG-PDLA	Cancer therapy	[49]

Lipid-based nanoparticles are nanoscale spherical platforms composed of at least one layer of lipids. These particles have several advantages, including high biocompatibility, protection of sensitive encapsulated agents, simple formulation, and capacity for targeted delivery. In addition, the physiochemical properties of lipid-based nanoparticles can be tuned through modification of their structures and surfaces. As a result, lipid-based nanoparticles are the most common FDA-approved nanomedicines. Liposomes—one type of lipid-based nanoparticles—are the earliest nanoparticles used for biomedicine: their history can be traced back to 1965 [50]. Liposomes are vesicular structures composed of lipid bilayers, which can form spontaneously when phospholipid molecules are exposed to water [51]. This unique structure enables the encapsulation of a wide variety of both hydrophilic and hydrophobic diagnostic [52, 53] or therapeutic [54, 55] agents, offering protection from clearance by the body. Since liposomes can be easily taken up by the reticuloendothelial system, structural and surface modifications have been reported to improve the efficiency and widen the applicability of liposomes [56]; liposomes have been used for environmental sensing [57–62], specific active targeting[63–65], and long circulation [66–68]. Applications of liposomes are currently limited by low loading capacity and fixed release kinetics [69, 70]. Lipid nanoparticles (LNPs) are primarily comprised of cationic ionizable lipids [71]. In contrast to the hollow core of liposomes, LNPs form a micelle structure with a solid core [72]. LNPs are typically composed of a mixture of lipids, which include ionizable cationic lipids to facilitate cellular uptake, phospholipids to form structures, cholesterol to improve stability, and polyethylene glycol (PEG)-lipids to provide steric stabilization [73]. The high stability, simple synthesis, high encapsulation efficiency, and strong transfection capacity make LNPs the gold standard for nucleic acid delivery [74]. Both FDA-approved mRNA vaccines for Covid-19 (Pfizer-BioNTech and Moderna) use LNPs as nanocarriers [41]. Applications of LNPs are currently limited by immunogenicity and uncontrolled biodistribution to organs other than the liver and spleen [75].

Polymer-based nanoparticles are synthesized from polymers, which are large molecules made of repeating monomers [71]. The properties of polymer-based nanoparticles—including release profiles, targeting, stability, responsiveness, and ability to encapsulate a wide range of agents [76]—can be precisely controlled by modulating polymer chemistry and particle composition [77]. Due to this tunability, polymer-based nanoparticles have gained significant attention in healthcare applications such as gene therapy [42], cancer therapy [45], and diagnosis [48]. Polymeric nanoparticles can be synthesized from either natural polymers—such as chitosan and Poly(L-lysine)—or synthetic polymers—such as Poly(lactide-co-glycolide), polylactide acid, and poly(caprolactone) [78]. Polymeric nanoparticles are widely researched for therapeutic applications due to their stability, tunable release kinetics, drug solubilization, and cellular uptake. Therapeutic agents can be either encapsulated, dissolved, entrapped, or attached to the polymer matrix and surface of these nanoparticles [79]. For instance, Blakney et al. reported a bioreducible, linear, cationic polymer, pABOL, for the delivery of self-amplifying mRNA that exhibits significantly less innate immunogenicity than traditional LNPs [44]. Applications of polymeric nanoparticles are currently limited by low drug loading efficiency and reproducibility [80]. Micelles are self-assembled nanostructures formed from amphiphilic block copolymers [71]. In aqueous environments, the hydrophobic blocks of the copolymer assemble at the core, while the hydrophilic blocks of the copolymer form the outer surface of the micelle [81]. This core–shell structure presents unique opportunities to encapsulate and deliver both hydrophobic and hydrophilic agents in the micelle’s hydrophobic core and hydrophilic shell, respectively [82]. The small size and high stability of micelles in blood circulation make them suitable for tumor targeting through the EPR effect [79]. Applications of micelles are currently limited by low stability and complex characterization [83]. A third type of polymer-based nanoparticles, dendrimers, are highly branched, tree-like macromolecules with a well-defined polymer structure [71] consisting of a central core, branching units, and terminal functional groups on each branch [84]. The size, shape, and surface chemistry of dendrimers can be highly controlled [71] to enable delivery of therapeutic and diagnostic agents. These agents can either be encapsulated in the internal cavities or be conjugated to the surface as functional groups via biodegradable linkers [85]. Applications of dendrimers are currently limited by high manufacturing costs and inherent toxicity[86]. We consider exosomes to be out of the scope of this review although acknowledge that they are also interesting nanomedicines [87, 88].

#### Inorganic nanomaterials

Inorganic nanomaterials are synthesized from inorganic precursors and can be precisely formulated with sizes and shapes ranging from 1 to 100 nm [72]. Representative nanomaterials in this class include metal and metal oxide nanoparticles[81]. These nanoparticles exhibit physical and chemical properties which are unique from their bulk material counterparts, due to their high surface to volume ratio and quantum confinement effects[89]. A summary of inorganic nanomaterials and their applications are shown in Table 2.

**Table 2:** Summary of common inorganic nanomaterials and their applications

Nanomaterials	Materials	Applications	Refs.
Metal nanoparticles	Gold, silver	Single molecule biosensor	[92]
	PEG, gold	Dual drug delivery	[91]
	SH-PEG, gold, silica	Near-infrared thermal therapy	[94]
Iron oxide nanoparticles	FeO(OH), oleic acid, 1-octadecene,	Magnetic resonance imaging	[98]
	Folic acid, Fe_3_O_4_, hyperbranched polyglycerol	Cervical cancer therapy, drug delivery	[99]
Metal organic frameworks	Mn, PEG-CDM-PEI	Cancer therapy	[7]
	ZIF-8	Glucose biosensor	[103]

Metal nanoparticles are nanoscale particles purely composed of metal precursors [90]. Metal nanoparticles composed of noble metals have been extensively studied due to their unique properties. For instance, gold exhibits excellent biocompatibility and remarkable optical characteristics [91], while silver demonstrates strong antibacterial activity and plasmonic properties [92]. Other metallic materials can offer characteristics such as magnetism and electrical activity. The unique properties of metal nanoparticles make them suitable for many applications, including diagnostic imaging[93], targeted drug delivery [91], photothermal therapy (PTT) [94], biosensing [92], and antimicrobial function [95]. Iron oxide nanoparticles, mainly composed of iron and oxygen, are another common type of inorganic nanoparticle [96]: in fact, the majority of FDA-approved inorganic nanomedicines are iron oxide nanoparticles [71]. Common forms include magnetite (Fe_3_O_4_) and maghemite (γ-Fe_2_O_3_), which possess natural magnetic properties and can be manipulated using external magnetic fields [97]. Due to their magnetic properties, iron oxide nanoparticles are widely used in MRI as contrast agents [98] and in hyperthermia for cancer treatment [99]. Applications of metal nanoparticles are limited by poor degradation, potential toxicity of heavy metals, and environmental risks [100, 101]. Metal organic frameworks (MOFs) [96] are another widely explored type of metallic-based nanoparticles. MOFs are hybrid materials that consist of metal ions or clusters embedded in a network of organic ligands, which form a regular lattice structure [102]. MOFs are well known for their high surface areas, tunable pore sizes, and versatile chemical functionalities [102]. Their high porosity results in high cargo loading capacity, which can be suitable for drug delivery [7] and sensing [103] applications. Currently, a variety of MOFs have been developed, including ZIFs [103], UIO-66 [104], Cu-MOF [105], and Fe-MOF[106]. Applications of MOFs are limited by instability in aqueous and biological environments [12].

#### Nanomaterial properties

Nanocarrier properties such as size, shape, surface charge, and surface chemistry [81] significantly affect their in vivo performance, including target site accumulation, circulation, biodistribution, cellular uptake, and toxicity [107].

##### Size and shape

Nanoparticles smaller than 10 nm typically are rapidly cleared from the bloodstream via renal filtration [108]. Larger nanoparticles can remain in circulation longer but may be taken up by the liver and spleen [108]. Nanoparticle size also affects cellular uptake: for example, Wu et al. reported that the size of silver nanoparticles influences not only the efficiency of cellular uptake, where uptake is most efficient for 100 nm diameter particles, but also the type of endocytosis through which silver nanoparticles enter cells [109]. Additionally, smaller nanoparticles exhibit higher toxicity than larger nanoparticles [101]. Further, nanoparticles with non-spherical shapes (e.g. stars or rods) may exhibit different biodistribution and cellular uptake pathways than spherical nanoparticles [110]. Shape can also influence the ability of nanoparticles to evade the immune system and interact with cell membranes [110].

##### Surface charge

Positively charged nanoparticles tend to demonstrate higher cellular uptake due to the negative charge on cell membranes, but also exhibit higher non-specific binding and potential toxicity [111]. Analogously, negatively charged or neutral nanoparticles can have prolonged circulation time and reduced non-specific cellular uptake. Wang et al. reported a charge-conversional click polyprodrug nanomedicine system, which can be negatively charged at pH 7.4 in the blood circulation to achieve prolonged circulation time and convert to be positively charged at pH 6.5 in the tumor microenvironment to increase cell binding [112].

##### Hydrophilicity and hydrophobicity

Hydrophobic nanoparticles are frequently rapidly cleared from the circulation by the immune system, while hydrophilic nanoparticles usually exhibit longer circulation times and lower protein absorption [113]. For example, Reboredo et al. developed zein nanoparticles for oral drug delivery, which are coated by PEG to increase the hydrophilicity of nanoparticles and thus improve their mobility in intestinal mucus [114].

### Applications and challenges

#### Applications of nanomaterials

Nanomaterials have made a significant impact on the medical field by offering innovative solutions to address challenges for multiple applications. In the following section, we discuss current research directions.

##### Applications in drug delivery

**Fig. 3:**
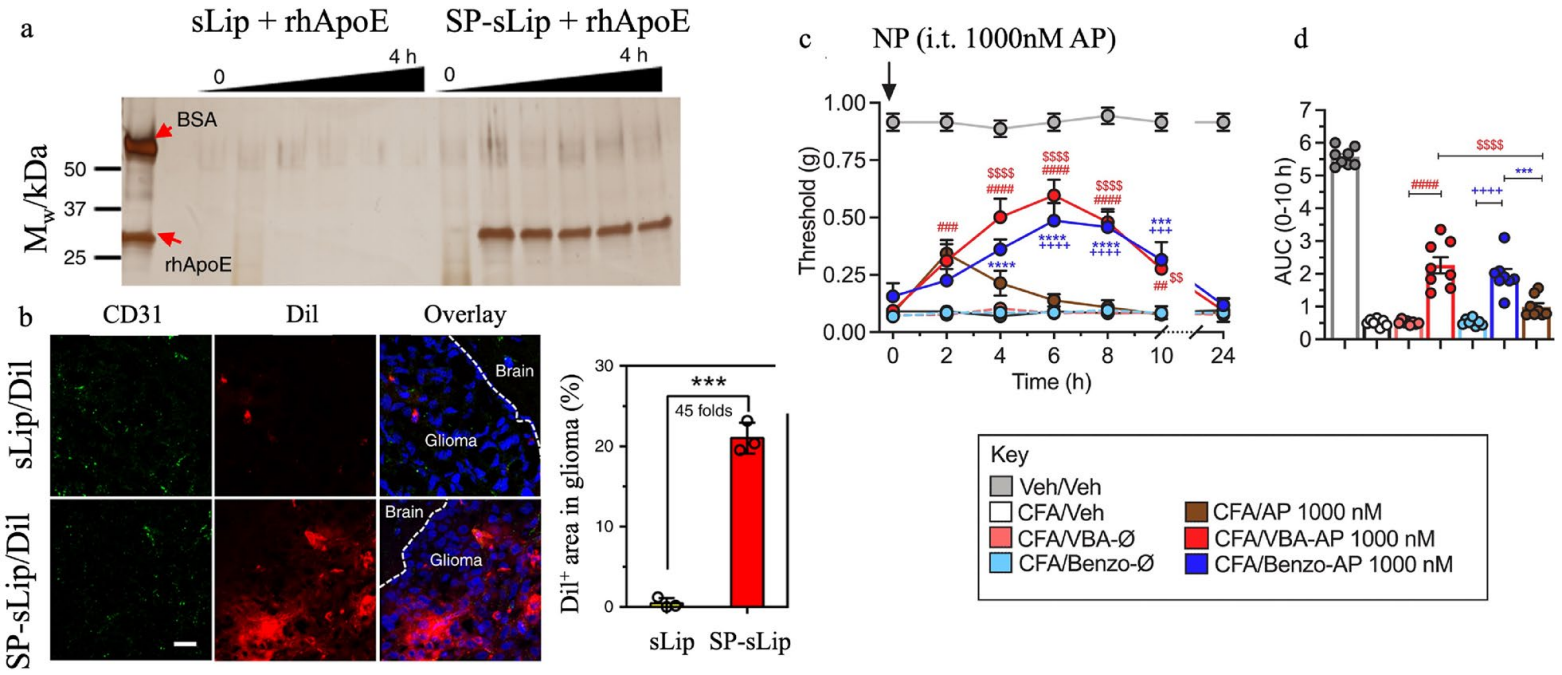
Applications of nanomaterials in drug delivery. a. Binding capacity and kinetics of recombinant human ApoE (rhApoE) on modified (SP-sLip) and unmodified (sLip) liposomal surfaces. **b** Biodistribution of modified and unmodified liposomes in intracranial glioma. Adapted with permission[37]. Copyright 2019, Springer Nature. c. Time course of effects of AP-nanostars and free AP on mechanical allodynia after intrathecal injection. d. Effective pain relief of AP at 10 h. Veh: vehicle, control. CFA: an emulsion of Complete Freund’s Adjuvant. VBA: 3-vinylbenzaldehyde. Benzo: 4-benzoylphenyl acrylate. Adapted with permission[115]. Copyright 2022, Elsevier

Nanoparticles can be engineered to deliver drugs specifically to diseased cells, thus minimizing side effects and improving therapeutic efficacy. One strategy to realize targeted drug delivery is to modify the surface of nanoparticles with peptides. For example, Zhang et al. modified the surface of liposomes with Aβ_1-42_ peptide to enable absorption of plasma apolipoproteins (i.e., ApoE, ApoJ, and ApoA1) onto the liposomal surface; the absorbed plasma apolipoproteins can then bind to the blood–brain-barrier transport protein LRP1 (Fig. 3a). Although unmodified liposomes can also bind to LRP1, the peptide-modified liposomes showed significantly higher uptake and distribution in intracranial glioma, as shown in Fig. 3b [37]. Nanoparticles can also encapsulate drugs, produce a controlled drug release profile, and ensure sustained drug delivery. Latorre et al. developed an aprepitant (AP) -nanostar sustained release system as a potential solution to chronic pain. Nanostars can sustain drug release for 24 h and thus maintain analgesia for more than 10 h (Fig. 3c and d). AP-nanostars treated mechanical and thermal allodynia more efficiently than free AP in preclinical models of neuropathic and inflammatory pain [115].

##### Applications in cancer therapy

**Fig. 4:**
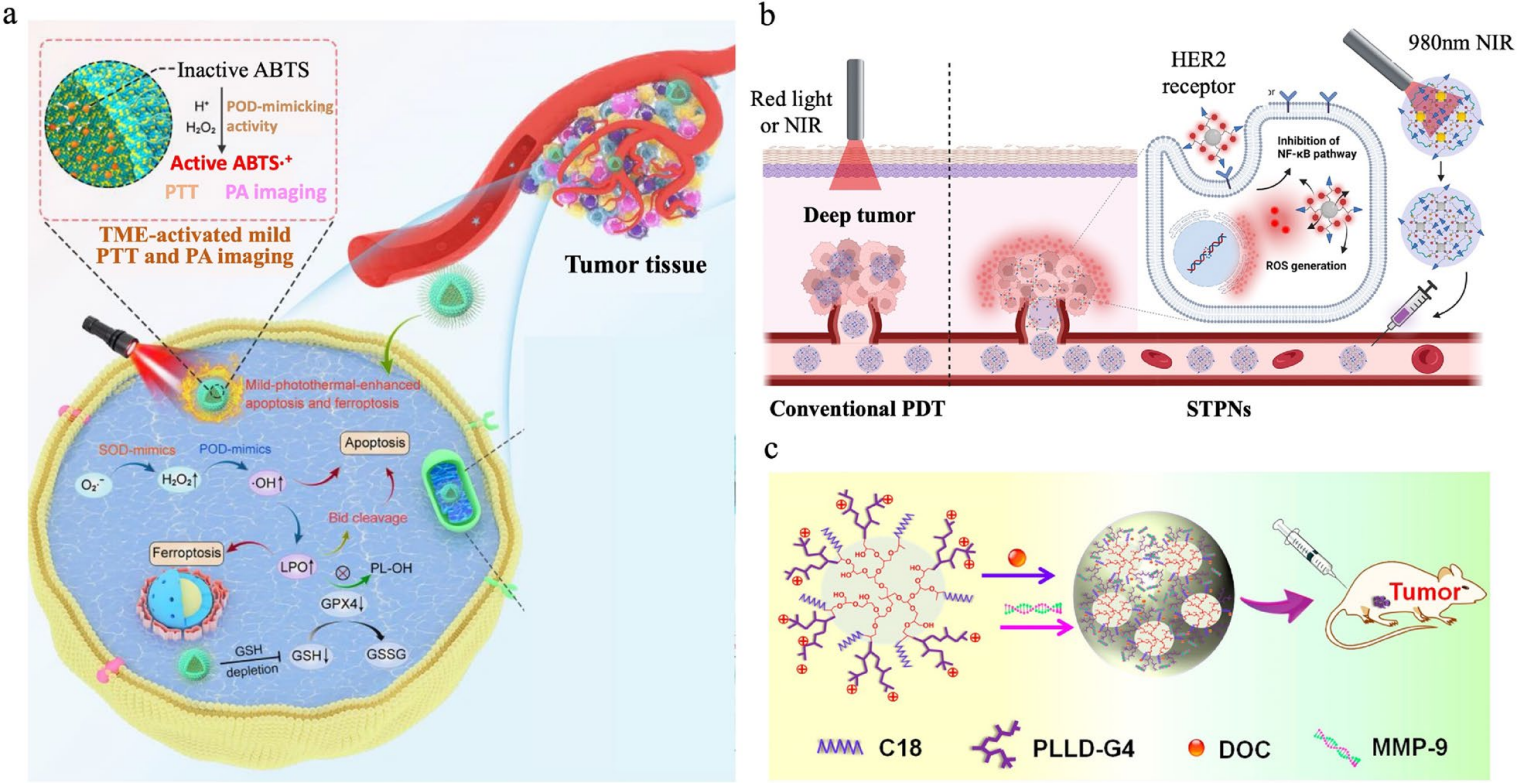
Applications of nanomaterials in cancer therapy. a. Schematic of the anticancer mechanisms of apoptosis and ferroptosis. POD: peroxidase. SOD: superoxide dismutase. LPO: phospholipid peroxidation. GSH: glutathione. GPX4: ferroptosis-related protein. TME: tumor microenvironment. Adapted with permission [116]. Copyright 2023, American Chemical Society. b. The difference between the mechanisms of STPNs activation for deep tumor therapy and conventional PDT. Adapted with permission [117]. Copyright 2023, Springer Nature. c. Schematic illustration of the synthetic process and administration process of the star-shaped copolymers for gene therapy. Reproduced with permission [47]. Copyright 2016, American Chemical Society

Nanomaterials have been reported to be useful for several cancer therapies, including PTT, PDT, and gene therapy. For PTT, nanomaterials can convert light into heat to selectively destroy cancer cells. Dong et al. developed mitochondria-targeting nanozymes, which perform intrinsic enzyme-like activities, to prevent the tolerance of temperatures up to 5 °C higher than body temperature often observed in tumor cells. When stimulated with near infrared (NIR) light, these nanozymes can mimic peroxidase activity and thus catalyze H_2_O_2_ found in the tumor microenvironment to form toxic ⋅OH and convert loaded 2,2′-azino-bis(3-ethylbenzothiazoline-6-sulfonic acid) (ABTS) to its oxidized form ABTS^+^, resulting in more precisely located PTT (Fig. 4a) [116]. For PDT, nanomaterials can produce reactive oxygen species (ROS) upon light activation to kill cancer cells. To overcome the limited tissue penetration of NIR, Juengpanich et al. developed stimuli-sensitive tumor-targeted photodynamic nanoparticles (STPNs). Before administration, STPNs can be excited by NIR irradiation and store the energy through the persistent luminescence of Purpurin 18 (Pu18). As presented in Fig. 4b, following stimulation of STPNs by the acidic tumor microenvironment, the nanoparticles disassembled and the photoactivity of Pu18 generated ROS to kill gallbladder cancer cells [117]. For gene therapy, nanomaterials can deliver genetic material to cells to treat genetic disorders and cancer. To induce apoptosis of cancer cells, Zhou et al. developed a star-shaped copolymer composed of amphiphilic octadecane-modified hyperbranched polyglycerol (C18) and poly(L-lysine) dendrons (PLLD). MMP-9 siRNA was loaded onto the poly(L-lysine) dendrons and the particles subsequently induced expression of MMP-9 in MCF-7 cells, yielding significant apoptosis of these cancer cells (Fig. 4c) [47].

##### Applications in imaging and diagnostics

**Fig. 5:**
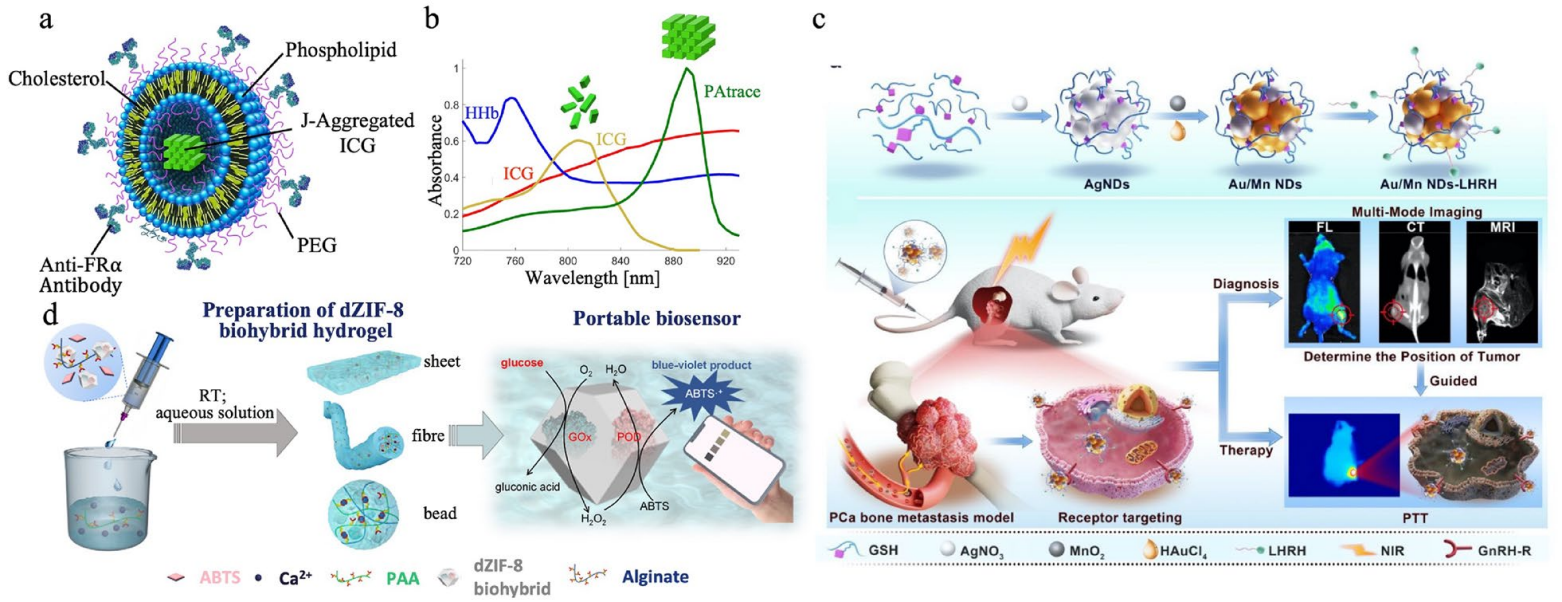
Applications of nanomaterials in imaging and diagnostics. a. Structure of PAtrace. b. Spectral feature of PAtrace compared with ICG, deoxyhemoglobin (HHb), and oxyhemoglobin (HbO_2_). Adapted with permission[38]. Copyright 2021, Springer Nature. c. Schematic of the theragnostic Au/Mn nano-system with multi-mode targeted imaging. Reproduced with permission [118]. Copyright 2023, Elsevier. d. Preparation process and schematic of the portable MOF hydrogel glucose sensor. PAA: poly(acrylic acid). Adapted with permission [103]. Copyright 2022, American Chemical Society

Nanomaterials can enhance the contrast in clinical imaging modalities such as MRI, CT, and ultrasound. Wood et al. introduced a photoacoustic (PA) contrast agent, PAtrace, which is based on J-aggregated indocyanine green (ICG) dye encapsulated in liposomes. PAtrace overcomes current limitations of PA contrast agents (Fig. 5a), including overlap of absorbance spectra and poor PA imaging sensitivity. Moreover, PAtrace has a sharp spectral feature around 890 nm, which allows for higher detection sensitivity in the presence of hemoglobin, as presented in Fig. 5b [38]. Nanomaterials can also enable simultaneous imaging using different techniques, providing comprehensive diagnostic information. For example, prostate cancer (PCa) is usually detected by MRI, but pinpointing the locations of metastases necessitates more complicated diagnostic techniques. Wang et al. reported a theragnostic Au/Mn nano-system with multi-mode targeted imaging which can be used for both CT/MRI and fluorescence visualization navigated surgery, as shown in Fig. 5c. The targeted agent, Luteinizing Hormone-Releasing Hormone (LHRH), was connected to the nano-system to provide an efficient solution for precise diagnosis of metastatic PCa [118]. Gonadotropin-Releasing Hormone Receptor (GnRH-R), the target receptor of LHRH, is highly expressed on the surface of PCa cells. Additionally, nanomaterials can enhance the sensitivity and specificity of biosensors for specific biomolecules. To develop a portable glucose biosensor, Zhong et al. encapsulated enzymes, consisting of glucose oxidase and peroxidase, into a defective MOF, ZIF, which was then double crosslinked by alginate hydrogel [103]. This ZIF can preserve the catalytic function of enzymes, which convert glucose into a blue-violet product, ABTS^+^ (Fig. 5d).

#### Challenges

##### Physical barriers

Nanoparticles face many physical barriers during administration to the human body—including the skin, mucosal surfaces, blood–brain barrier, and tumor stroma—which significantly influence their biodistribution, cellular uptake, and therapeutic efficacy. First, nanoparticles encounter the skin, which consists of the epidermis, dermis, and hypodermis [119]. The topmost sub-layer of epidermis, the stratum corneum, prevents the penetration of foreign substances, including nanoparticles, and limits the rate of diffusion [31]. Transdermal delivery strategies overcome this barrier by designing nanoparticles with certain size, shape, and surface properties or employing physical methods (e.g., microneedles) [30]. Next, the endothelial barrier, which is formed by the endothelial cells lining the blood vessels, regulates the passage of nanoparticles between the bloodstream and tissues [31]. The tight junctions between endothelial cells restrict the paracellular transport of nanoparticles [120]. Then, the EPR effect in the tumor vasculature allows nanoparticles to preferentially accumulate in tumor tissue, though the efficiency of the EPR effect varies across patients and cancer types [121]. Additionally, the mucosal surfaces found in the gastrointestinal and respiratory tracts are composed of a layer of mucus; this viscous and sticky gel can trap and remove nanoparticles [122]. To overcome the mucosal barrier, nanoparticles can be coated with polymers to enhance retention or augmented with physical methods [122]. The extracellular matrix (ECM) can also significantly impact the penetration of nanoparticles. For example, the fibrotic tissue formed in some diseases has a higher ratio of collagen than normal ECM, which forms a dense network that restricts the penetration, diffusion, and biodistribution of nanoparticles [31, 121]. This problem can be addressed by optimizing the size, shape, and surface properties of nanoparticles or supplementing with external stimuli to aid the penetration of nanoparticles [123].

##### Biological barriers

The Mononuclear Phagocyte System (MPS), also known as the reticuloendothelial system, consists of immune cells (primarily macrophages) in the liver, lung, and spleen. The MPS can identify, capture, and eliminate foreign nanoparticles from the bloodstream [123, 124]. In brief, after administration, nanoparticles sequestered by the MPS adsorb plasma proteins onto their surface, including serum albumin, apolipoproteins, complement components, and immunoglobulins [123]. Following protein adsorption, nanoparticles can be recognized by specific receptors on the surface of phagocytes and subsequently rapidly cleared from the body [125]. This process limits the circulation time of nanoparticles, reducing the probability that they reach their target site and perform their biomedical function. Strategies to evade the MPS include modifying the nanoparticle surface with PEG or other "stealth" materials to reduce protein adsorption and thus delay recognition and clearance of nanoparticles by immune cells [126].

##### Patient adherence

Nanoparticles face challenges related to patient adherence to therapeutic regimens. Nanoparticle-based therapies are often delivered by injection administered by medical experts. This complex delivery method can discourage consistent use of nanoparticles, especially by patients who have needle phobias or lack access to healthcare facilities. Moreover, nanoparticles with complex structures may induce unintended biological responses, including toxicity or systemic immune reactions [127], which can result in apprehension towards and discontinuation of the therapy by patients. Additionally, the production of nanoparticle-based therapies involves expensive techniques [18], resulting in financial burdens which further reduce patient adherence.

## Strategies for microneedle-assisted nanomedicine

### Microneedle technology

Microneedles are microscale needle-like structures designed to deliver therapeutic and diagnostic agents. Microneedle technologies have attracted growing interest for biomedical applications such as biosensing, health monitoring, and drug delivery due to their minimally invasive design and dimensions which avoid triggering nerves and thus minimize patient pain [128]. Additionally, microneedle devices have the potential to reduce or eliminate the need for expert supervision [129], the production of biohazardous sharps waste, and the risk of needle stick injury [130]. Further, microneedles enable loaded substances to be directly delivered to target positions and precise regulation of dosage [32]. Microneedles can be designed to ensure efficient drug penetration and delivery while minimizing patient pain and discomfort through careful consideration of factors such as length, diameter, and shape. Microneedles can be further organized into integrated arrays on a single patch to achieve multiple functions [131].

**Fig. 6:**
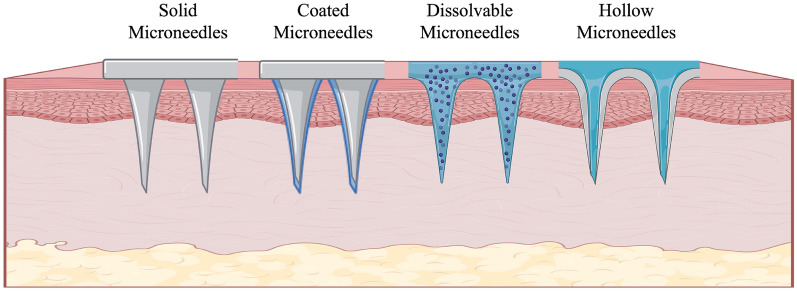
Representative types of microneedles, including solid microneedles, coated microneedles, dissolvable or degradable microneedles, and hollow microneedles

As illustrated in Fig. 6, microneedles can be divided into four types: solid; coated; dissolvable or degradable; and hollow [132]. Solid microneedles are typically constructed of metal and silicon and are not loaded with agents [133]; their function is to enhance permeability for subsequent applications. Coated microneedles are primarily designed for drug delivery; they can produce rapid drug release profiles for immediate therapeutic effects [134]. Dissolvable or degradable microneedles can eliminate sharps waste, enhancing safety and compliance. This type of microneedle is suitable for localized drug delivery and can be used for controlled release of diagnostic agents for allergy testing or other diagnostic purposes [134]. Hollow microneedles enable the delivery of liquid agents and establish precise control over drug dose and release profile [134]. A summary of microneedle types and their applications is shown in Table 3.

**Table 3:** Summary of common microneedle types and their applications

Microneedles	Materials	Applications	Refs.
Solid microneedles	Silicon	Targeted epidermal delivery	[135]
	Titanium	Transdermal drug delivery	[136]
	Silicon, aurum	Transdermal glucose monitor	[137]
	Silicon, aurum	Breast cancer biomarker detection	[138]
Coated microneedles	Microneedles: titanium Coated layer: desmopressin	Transdermal drug delivery	[139]
	Microneedles: silicon Coated layer: recombinant adenovirus and modified vaccinia virus Ankara	Transcutaneous virus delivery	[140]
	Microneedles: stainless steel (304) Coated layer: 5-aminolevulinic acid	Photodynamic therapy	[141]
	Microneedles: poly(L-lactide) Coated layer: peptide nucleic acid-alginate	Skin interstitial fluid sensor	[142]
Dissolvable microneedles	Poly(lactic-co-glycolic acid) (PLGA)	Electrotherapy and drug delivery	[143]
	Gelatin methacryloyl	Intestinal macromolecule delivery	[144]
	Hyaluronic acid (HA)	Transdermal drug delivery	[145]
	Polyvinylpyrrolidone (PVP)	Buccal macromolecule delivery	[146]
Hollow microneedles	Nickel	Insulin delivery	[147]
	Silicon	Hydrodynamic gene delivery	[148]
	PLGA	Transdermal vaccine delivery	[149]
	Poly(ethylene glycol) diacrylate (PEGDA)	Dermal interstitial fluid sensor	[150]

**Fig. 7:**
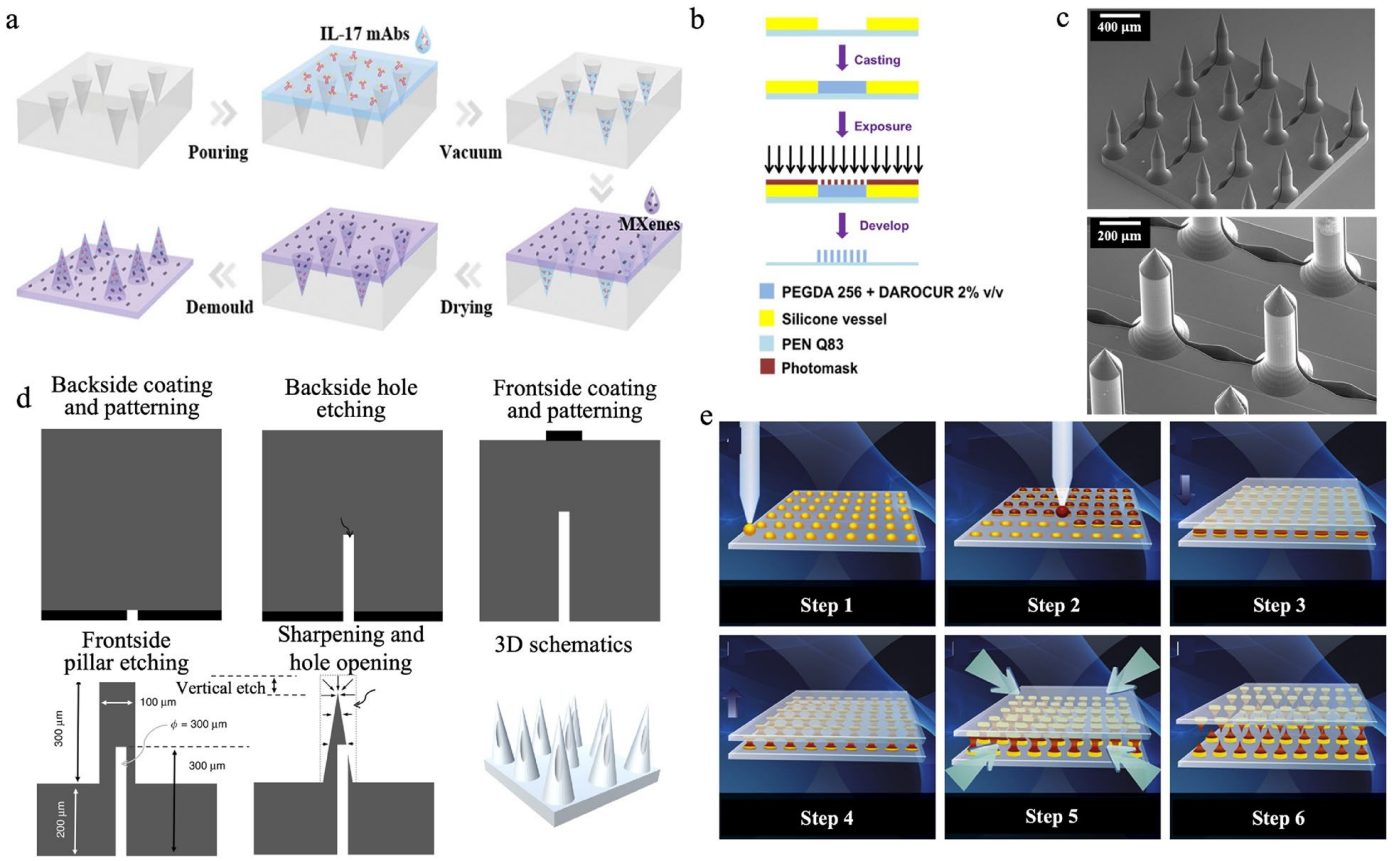
Methods for fabrication of microneedle structures. a. Manufacturing process of microneedle patches using the micromolding method, including pouring, vacuum, drying, and demold. Reproduced with permission [152]. Copyright 2022, Wiley–VCH GmbH. b. Schematic of fabrication of microneedle structures using the photolithography method with a mixture of two photoresists (PEGDA and DAROCUR), a silicone vessel, a hard sheet of plastic-like acetate (PEN Q38), and a photomask, initiated by casting. Reproduced with permission [153]. Copyright 2015, CC-BY. c. SEM image of a TPP fabricated microneedle structure with open microfluidic channels. Adapted with permission [155]. Copyright 2017, Springer Nature. d. Fabrication process of silicon hollow microneedle arrays, followed by backside hole etching and frontside pillar etching. Adapted with permission [156]. Copyright 2019, Springer Nature. e. Schematic of microneedle fabrication using the DAB method with key steps including droplet dispensing, contact, length control, and air blowing. Adapted with permission [157]. Copyright 2013, Elsevier

Microneedles can be made from both active and passive materials. Active materials can change their properties in response to external stimuli (e.g., temperature, pH, and light) and thus allow for controlled release profiles and enhanced functionalities. Passive materials maintain stable and reliable structures despite external stimuli and are generally considered to be both biocompatible and safe for biomedical use [151]. Currently, microneedles can be fabricated using several methods, including micromolding, photolithography, 3D printing, etching, and droplet-born air blowing (DAB). Micromolding involves casting materials into microneedle-shaped molds; this technique is suitable for mass production because it is relatively simple and cost effective. As illustrated in Fig. 7a, Wu et al. fabricated a photothermally dissolvable microneedle patch using micromolding. First, a solution of IL-17 monoclonal antibodies (mAbs) was poured into the microneedle mold; then, a sodium hyaluronate hydrogel encapsulating MXene, a 2D biodegradable niobium carbide material, was cast onto the mold. The hydrogel containing mAbs and Mxene filled the mold by vacuum; after drying, the microneedle patch was easily removed from the mold [152]. Photolithography uses light to pattern photosensitive materials and can fabricate microneedle shapes through exposure of photosensitive material to UV light through a photomask. As shown in Fig. 7b, Dardano et al. used polyethylene naphthalate (PEN) as the microneedle substrate and put the photosensitive material PEGDA into a silicone vessel. The PEGDA was exposed to UV light through a photomask to create the microneedle structure [153]. 3D printing technologies—such as fused deposition modeling, stereolithography, digital light processing, and two photon polymerization (TPP)—allow for the creation of intricate shapes and structures and can be directly adopted to fabricate microneedles [154]. Rad et al. fabricated microneedle patches with open microfluidic channels by TPP 3D printing, as presented in Fig. 7c[155]. Initially, a 3D model of the microneedle structure was generated and imported to the software Describe for setting the printing parameters. Then, the laser beam was immersed in IP-S, a photoresist, to print the microneedle structure layer by layer. Etching involves selectively removing material to form microneedle shapes; this process is mainly used to create silicon or metal microneedles. As presented in Fig. 7d, Li et al. used anisotropic etching of silicon structures to fabricate hollow microneedle arrays [156]. The DAB method involves elongating droplets of polymer solution, which are then solidified by blowing air to create microneedle structures. Kim et al. fabricated a microneedle patch using the DAB method, where carboxymethylcellulose (CMC) was the base material and a mixture of CMC, sodium hyaluronate, and PVP was the microneedle material (Fig. 7e) [157]. Advantages and disadvantages of microneedle fabrication methods are summarized in Table 4.

**Table 4:** Summary of common microneedle fabrication methods

Fabrication methods	Advantages	Disadvantages	Refs.
Micromolding	Simple and cost effective; reusability of molds	Demolding challenges; mold fabrication complexity; batch variation	[152, 158–161]
Photolithography	One-step simple fabrication process	Blunt needle tips; material limitations	[153, 162, 163]
3D printing	Direct fabrication; high printing resolution; intricate and precise structure; customizable design	Expensive equipment; equipment-dependent resolution; slow printing speed	[133, 155, 164–167]
Etching	High precision; complex structure fabrication	Rough surface; complicated fabrication process; environmental concerns; difficulty in integrating drug loading	[156, 168–170]
DAB method	Drug activity; low cost; simple equipment; material versatility	Limited microneedle structure complexity; limited scalability; environmental sensitivity	[157, 171, 172]

### Development of microneedle-based delivery systems

Microneedle technologies are advancing drug delivery systems by providing patient-centric approaches for the administration of various therapeutic agents. Microneedle-based delivery systems can support multiple drug release mechanisms. Passive drug release can be achieved through the dissolution of drug-loaded microneedles [146] or the diffusion of coated drugs on the surface of microneedles [173] for consistent and controlled delivery. Active drug release can be precisely controlled by external stimuli, such as electrical currents [174] and ultrasonic waves [145], to enhance drug permeation and release. Responsive drug release can be achieved by fabricating microneedles using materials which respond to specific physiological signals (e.g., pH [175], temperature [176], or biomolecules [177]) to trigger drug release.

In addition, microneedle technologies have demonstrated potential in the field of theragnostics, including as open-loop systems comprised of independent diagnosis [178] and therapy [179] units and as closed-loop systems that offer personalized and adaptive treatment based on continuous monitoring of physiological status [180]. Closed-loop systems are particularly promising for treating chronic diseases [181], due to their capacity to enhance therapeutic outcomes and minimize overdosing side effects.

Furthermore, microneedle systems can accommodate diverse therapeutic and diagnostic needs by delivering cargoes ranging from small molecules [182] and biologics [183] to vaccines [184] and diagnostic agents [185]. Microneedles are also compatible with multiple administration approaches. Topical administration offers a non-invasive and convenient approach for applications such as transdermal drug delivery [186], vaccination [187], and wearable monitoring [188]. Oral administration can enhance the bioavailability of drugs with poor oral absorption, ensuring sufficient therapeutic outcomes [189]. Implantable microneedle systems can provide sustained drug release and continuous monitoring [143], so are ideal for long-term therapy of chronic diseases.

#### Advantages of microneedle-based delivery systems

Microneedle-based delivery systems have gained significant attention owing to their advantages for diagnostic and therapeutic applications. Most microneedle devices are still in clinical trials or on the market without FDA approval. SkinPen is the first FDA-cleared microneedle device; it is clinically proven to be safe and effective for treatment of facial acne scars on all types of skin in patients aged 22 and over[190]. The main advantages of microneedle-based delivery systems are as follows:

**Patient adherence.** For transdermal delivery, microneedles only penetrate the outermost layer of the skin to ensure minimally invasive administration, which reduces pain and discomfort and enhances patient adherence to treatment [191]. Also, microneedle patches can be designed for easy self-administration, offering patients the option to manage their health and treatment without expert manipulation [192]. This possibility is particularly beneficial to those who suffer from chronic disease and require regular treatment [193]. Microneedle fabrication materials can also be designed and selected to realize controlled and sustained drug release, thus reducing the frequency of dosing [192]. In addition, the minimally invasive property of microneedle systems minimizes the risk of infection compared with traditional injections [194].

**Enhanced drug absorption.** By penetrating the stratum corneum of skin and the epithelial cell layer of various tissues, microneedles facilitate the absorption of therapeutics by target tissues and thus improve therapeutic outcomes [195].

**Targeted and versatile delivery.** Microneedles can be placed in specific sites of the human body to precisely deliver cargo to target tissues and minimize side effects. Additionally, microneedles are compatible with multiple types of cargoes, including small molecules[182], biologics[183], vaccines [184], and diagnostic agents [185].

**Cost effectiveness.** Microneedle systems can be mass-produced and reduce healthcare costs by minimizing the need for professional administration and reducing hospital visits [196].

#### Challenges of microneedle-based delivery systems

Microneedle-based delivery systems have shown significant potential to administer various cargoes, but these systems face some inherent challenges. Due to the limited surface area and volume of microneedles, their drug loading capacity is limited, especially for large biomolecules [2]. Additionally, it is difficult to maintain the stability of biologics within microneedles during device fabrication and storage [197]. For example, biologics stability is crucial when microneedles are used to deliver vaccines, because maintaining antigen integrity is essential to immunogenicity [198], and when microneedles are used in diagnostic applications, which rely on the efficient capture and detection of biomarkers [199].

To address these challenges, microneedle technologies can be integrated with nanomedicine. Nanoparticles have the capacity to encapsulate and concentrate therapeutic agents [200], thereby expanding microneedles' drug loading capabilities. Moreover, nanoparticles as nanocarriers provide a protective environment for sensitive molecules [201], ensuring their stability and activity. Nanomedicine systems can be designed for controlled release to ensure consistent and prolonged drug release via microneedles. They can also facilitate enhanced penetration of therapeutic agents through diffusion across the endothelium and epithelium or interaction with receptors expressed in the target area, ensuring efficient delivery to the target site [202]. Furthermore, nanomedicines can serve as adjuvants [203], improving the immunogenicity of vaccines administered via microneedles. Nanomedicine can also improve the sensitivity and specificity of diagnostic agents [204], ensuring that microneedle-based diagnostic systems can efficiently capture and detect biomarkers.

Thus, the combination of microneedle technologies with nanomedicine offers innovative solutions to overcome the challenges associated with single microneedle-based or nanomedicine-based cargo delivery, paving the way for advanced and optimized therapeutic and diagnostic interventions.

### Microneedle-mediated strategies to advance nanomedicine

The conventional delivery of nanomedicine, whether for therapeutic or diagnostic purposes, encounters specific challenges that can be effectively addressed through the integration of microneedle-based delivery systems.

#### Therapeutic applications

**Fig. 8:**
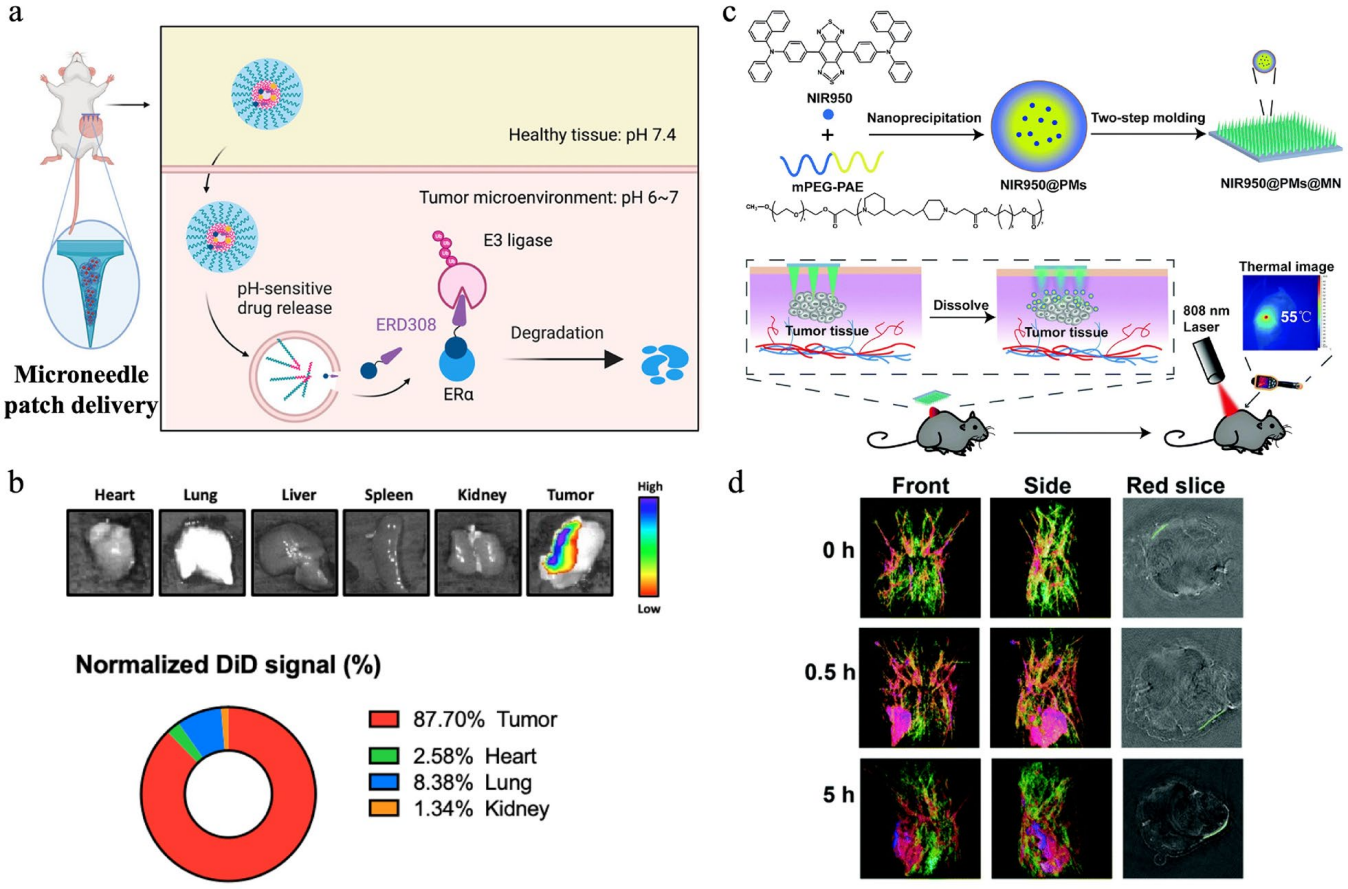
Microneedle-mediated nanomedicine for cancer therapy. a. Schematic of PROTAC microneedle delivery system. b. Biodistribution of PROTAC encapsulated micelles. Adapted with permission [205]. Copyright 2023, American Chemical Society. c. Schematic of fabrication and administration of NIR950 loaded microneedle patches. d. In vivo optoacoustic imaging of tumor-bearing mice at different time points after administration of NIR950 loaded microneedle patch. Reproduced with permission [206]. Copyright 2020, Royal Society of Chemistry

Nanomedicine for delivery of cancer therapies is often hindered by limited drug penetration into solid tumors, leading to poor efficacy and systemic toxicity. Microneedles can address this problem by precisely and directly delivering cancer-targeting nanomedicine into tumors and penetrating the ECM of tumors. Microneedle-mediated nanomedicine improves drug distribution and reduces systemic exposure, thereby improving therapeutic outcomes. Cheng et al. reported a microneedle delivery system to achieve sustained release of proteolysis-targeting chimeras (PROTACs), which degrade disease-related proteins. To treat breast cancer, a PROTAC targeting estrogen receptor alpha (ERD308) was encapsulated into pH-sensitive micelles, which were subsequently mixed with methacrylated HA to fabricate microneedle patches (Fig. 8a). These patches provided sustained release into deep tumors, where over 87% of the drug was retained in the tumors (Fig. 8b) [205]. Microneedles can also enable rapid accumulation of photothermal agents. Wei et al. fabricated microneedle patches using PVP/ polyvinyl alcohol (PVA) and encapsulated NIR950-loaded pH-sensitive micelles for melanoma photothermal therapy (Fig. 8c). NIR950 offers photostability and high photothermal conversion efficiency but has a long circulation period so requires 24 h to accumulate in therapeutic quantities at tumors when administered through intravenous injection. With the help of a microneedle patch, a strong NIR950 signal was detected at the tumor after 0.5 h, as shown in Fig. 8d [206].

**Fig. 9:**
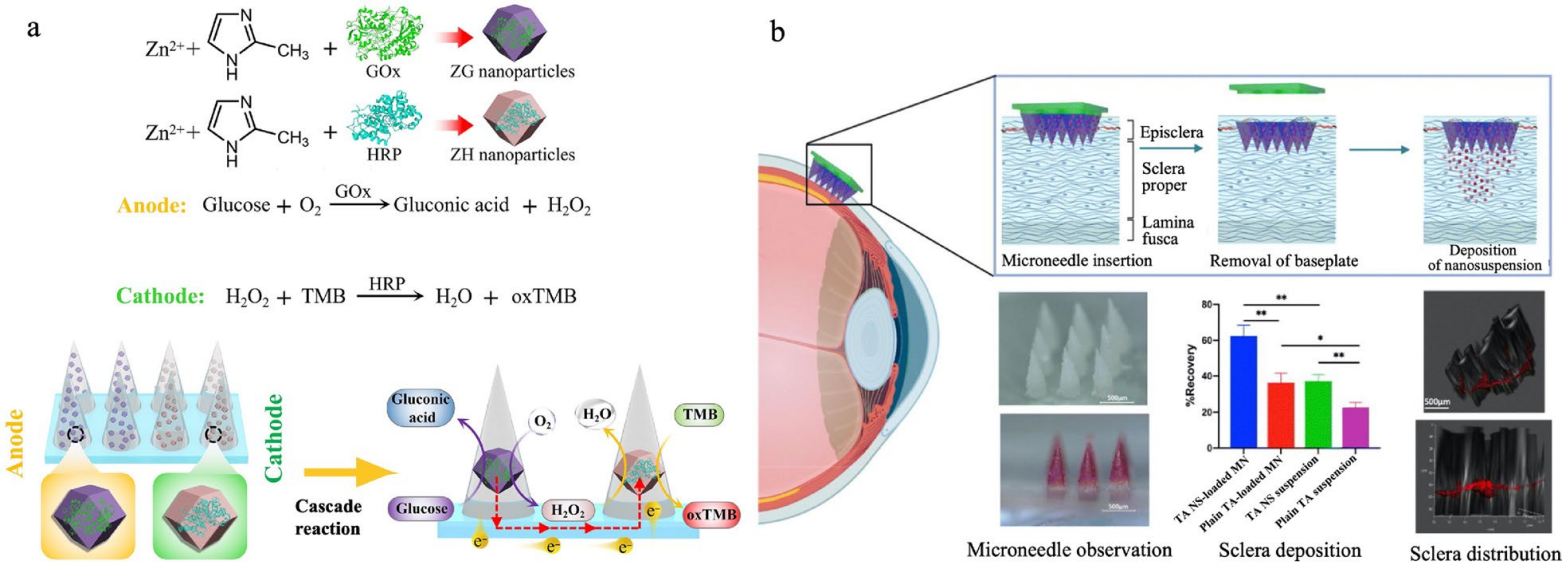
Microneedle-based nanomedicine for dermatological treatments and ocular drug delivery. a. Schematic of fabrication and administration of a self-powered enzyme-linked microneedle patch. TMB: 3,3’,5,5’-tetramethylbenzidine. Adapted with permission [207]. Copyright 2023, CC BY-NC. b. Schematic and therapeutic outcome of a nanosuspension-loaded dissolving microneedle system. Adapted with permission [208]. Copyright 2022, Elsevier

For dermatological treatments, traditional nanomedicine can struggle with uneven drug distribution within the skin layers, but microneedles create microchannels in the skin, which ensure uniform drug delivery and improve treatment results. Diabetic wounds can be difficult to heal due to their complex pathological environment. To address this challenge, Zhang et al. designed a self-powered enzyme-linked microneedle patch made of HA/PVA and encapsulating MOFs, ZIF-8 [207]. Inspired by the hypothesis that the loss of bioelectricity might be a primary reason diabetic wounds fail to heal, this microneedle comprises an anodic part and a cathodic part to stimulate bioelectricity. The ZIF-8 in the anode contains glucose oxidase (GOx), while the ZIF-8 in the cathode contains horseradish peroxidase (HRP). As illustrated in Fig. 9a, GOx consumes glucose to generate electricity, while the oxygen produced by HRP aids in wound healing.

Similarly, ocular drug delivery faces challenges presented by limited residence time and frequent dosing. Intravitreal injections have always been considered as the gold standard to deliver drugs for the treatment of retinal diseases. However, their high invasiveness and severe side effects lead to poor patient compliance. Microneedles can overcome these challenges by offering sustained drug release within the eye, thereby improving efficacy and patient compliance. Wu et al. reported a microneedle delivery system encapsulating a nanosuspension (NS) to deliver the hydrophobic drug triamcinolone acetonide (TA)—an anti-inflammatory corticosteroid—to the eye, as illustrated in Fig. 9b [208].The microneedle patch, made of PVP/PVA, can dissolve rapidly upon reaching the target site, much like wearing contact lenses. Compared to plain TA as a control, TA-loaded microneedles exhibited the best sclera deposition, followed by similar performance by TA NS and TA NS-loaded microneedles (Fig. 9b).

#### Theragnostic applications

**Fig. 10:**
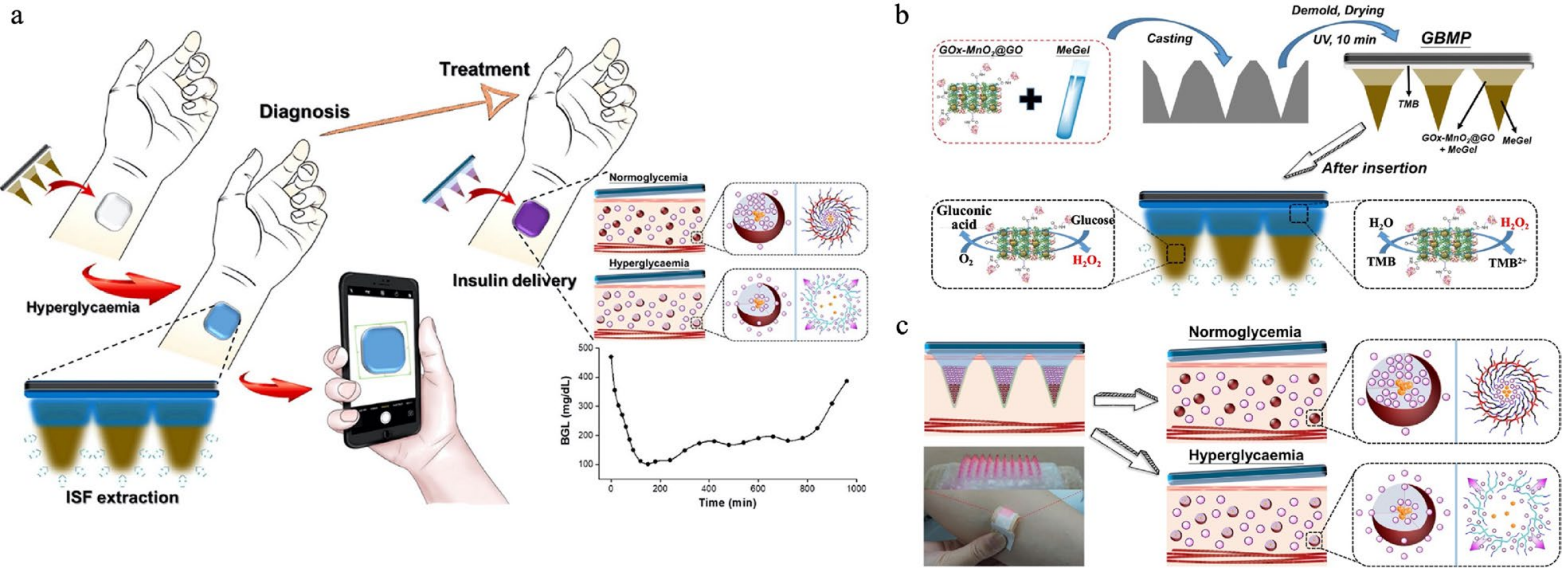
Microneedle-based nanomedicine for theragnostic applications. **a** Schematic of the on-skin glucose biosensor and on-demand insulin delivery. **b** Fabrication and administration process of GBMP. c. Schematic of administration of IDMP. Adapted with permission [181]. Copyright 2020, Elsevier

In the field of theragnosis, conventional blood sampling for diagnostic purposes can be painful and requires skilled personnel. Microneedles offer a painless alternative by enabling patients to collect small blood samples themselves for diagnostics and reducing reliance on healthcare professionals. This approach has potential applications in diabetes, which requires accurate monitoring of blood glucose levels in the human body and accounts for 11.3% of global deaths. The most common method for detecting glucose levels is the lancet diagnostic method, which is an invasive process that may cause pain and inflammation and is followed by two or three insulin injections every day, which may lead to lipodystrophy. Hsu et al. developed a theragnostic system comprised of two parts: a skin-mounted glucose biosensing microneedle patch (GBMP) and an on-demand insulin delivery microneedle patch (IDMP) (Fig. 10a)[181]. For the GBMP, GOx-conjugated MnO_2_/graphene oxide nanozymes were mixed with methacrylated gelatin to build the microneedle patch. When in contact with interstitial fluid, this patch produced gluconic acid and H_2_O_2_, the latter of which facilitated the oxidation of TMB (Fig. 10b). The combination of TMB and MnO_2_ changed the color of the patch from colorless to blue, which could be measured by a mobile phone. Then, IDMP was applied to sustainably deliver insulin. A branched poly (β-amino esters) (bPAEs) was used to manufacture nanovesicles to encapsulate insulin and GOx. These nanovesicles were then mixed with PVA/PVP to create the microneedle structure. Once inserted into the skin, the free insulin reduced the glucose level to normoglycemic levels. When the glucose level returned to hyperglycemia, the insulin encapsulated in the nanovesicles was released (Fig. 10c).

In conclusion, the integration of microneedle-based delivery systems with nanomedicine approaches has proven instrumental in addressing challenges related to drug distribution, drug stability, patient comfort, and treatment accessibility. Ultimately, this technology has the potential to revolutionize the field of diagnostics and therapeutics.

## Conclusions and outlook

Extensive research efforts have developed many nanomaterial systems, and the structure and function of these systems have become increasingly complex. Commonly used nanomaterials include those which are lipid-based, polymer-based, and inorganic. Despite their demonstrated potential for biomedical applications, nanomaterials still face challenges, such as rapid clearance by the liver, poor patient adherence, and limited passage through biological barriers. Microneedle structures have recently earned the attention of researchers due to their capacity for targeted delivery, high patient compliance, and easy fabrication. However, microneedle devices also have shortcomings, including insufficient penetration and limited drug loading capacity.

Microneedles and nanomaterials can complement each other when the technologies are integrated. For example, the penetration depth and targeting capacity of microneedles can enhance the therapeutic efficiency of nanomedicine. Meanwhile, nanomedicine can encapsulate, protect, and concentrate therapeutic agents. To realize the potential of the combination of microneedles with nanomedicine, some practical issues must be addressed. Specifically, incorporation of nanomaterials into microneedle structures may affect the mechanical performance of microneedles. Additionally, scalable and cost-effective manufacturing processes must be developed to enable translation of these systems to the clinic. Furthermore, the stability of nanodrugs combined with microneedles remains unclear. Lastly, microneedle-mediated nanomedicine systems lack clear regulatory guidelines and may thus require significant time and resources to obtain necessary approvals.
